# Genome-wide methylation analyses identifies Non-coding RNA genes dysregulated in breast tumours that metastasise to the brain

**DOI:** 10.1038/s41598-022-05050-z

**Published:** 2022-01-20

**Authors:** Rajendra P. Pangeni, Ivonne Olivaries, David Huen, Vannessa C. Buzatto, Timothy P. Dawson, Katherine M. Ashton, Charles Davis, Andrew R. Brodbelt, Michael D. Jenkinson, Ivan Bièche, Lu Yang, Farida Latif, John L. Darling, Tracy J. Warr, Mark R. Morris

**Affiliations:** 1 Research Institute in Healthcare Science, University of Wolverhampton, Wolverhampton, UK; 2 School of Science, University of Wolverhampton, Wolverhampton, UK; 3 Department of Neurosciences, Lancashire Teaching Hospitals NHS Foundation Trust, Royal Preston Hospital, Preston, UK; 4 The Walton Centre NHS Foundation Trust, Lower Lane, Liverpool, UK; 5 Department of Genetics, Institute Curie, Paris, France; 6 Department of System Biology, Beckman Research Institute, City of Hope National Medical Centre, Duarte, CA 91016 USA; 7 Institute of Cancer and Genomic Sciences, College of Medical and Dental Sciences, University of Birmingham, Edgbaston, Birmingham, UK; 8 Institute of Translational Medicine, University of Liverpool, Liverpool, UK; 9 Present Address: Division of Hematology/Oncology, Departments of Medicine, Northwestern University Feinberg School of Medicine, Chicago, USA; 10 Present Address: Department of Natural and Applied Sciences, Nexus Institute of Research and Innovation (NIRI), Lalitpur, Nepal; 11 Present Address: Molecular Oncology Centre, Laboratory of Bioinformatics, Hospital Sirio-Libanes, Sao Paulo, Brazil

**Keywords:** Cancer, Genetics, Molecular biology, Biomarkers, Molecular medicine, Oncology

## Abstract

Brain metastases comprise 40% of all metastatic tumours and breast tumours are among the tumours that most commonly metastasise to the brain, the role that epigenetic gene dysregulation plays in this process is not well understood. We carried out 450 K methylation array analysis to investigate epigenetically dysregulated genes in breast to brain metastases (BBM) compared to normal breast tissues (BN) and primary breast tumours (BP). For this, we referenced 450 K methylation data for BBM tumours prepared in our laboratory with BN and BP from The Cancer Genome Atlas. Experimental validation on our initially identified genes, in an independent cohort of BP and in BBM and their originating primary breast tumours using Combined Bisulphite and Restriction Analysis (CoBRA) and Methylation Specific PCR identified three genes (*RP11-713P17.4*, *MIR124*-2, *NUS1P3)* that are hypermethylated and three genes (*MIR3193*, *CTD*-*2023M8*.1 and *MTND6P4*) that are hypomethylated in breast to brain metastases. In addition, methylation differences in candidate genes between BBM tumours and originating primary tumours shows dysregulation of DNA methylation occurs either at an early stage of tumour evolution (in the primary tumour) or at a later evolutionary stage (where the epigenetic change is only observed in the brain metastasis). Epigentic changes identified could also be found when analysing tumour free circulating DNA (tfcDNA) in patient’s serum taken during BBM biopsies. Epigenetic dysregulation of *RP11-713P17.4*, *MIR3193*, *MTND6P4* are early events suggesting a potential use for these genes as prognostic markers.

## Introduction

More than 90% of cancer related deaths are attributed to metastases and about 80% of breast cancer deaths occur from metastases^1^. 15–25% of breast tumours metastasise to the brain during the course of disease^2^ and despite aggressive treatment strategies with surgical resection, stereotactic radiosurgery and whole brain radiotherapy (WBRT), the prognosis of breast to brain metastasis (BBM) patients remains poor^3^. Due to the challenges associated with the treatment of brain tumours, it is crucial to find novel prognostic markers that will inform the clinical management of breast cancer patients.

The seed and soil theory of metastases indicates that metastasing tumour cells have affinity for specific organs^4,5^. Metastases arise from metastatic initiating cells, which intravasate from the primary sites and remain as undetectable disseminated tumour cells before evolving into clinically visible lesions in distant organs^6^. These dormant cells are often found as micrometastases in bone marrow and lymph nodes many years after primary tumour treatment^7–9^.

Primary breast tumour hormone receptor status (Estrogen receptor (ER), Progesterone Receptor (PR) and Human EGFR Receptor 2 (HER2)) can be used as prognostic markers for brain metastasis risk^10,11^ and BBM occurs more frequenty in patients with ER–, HER2 + or triple negative breast tumours (ER-/PR-/HER2-)^12^. Of the different tumour subtypes, triple negative breast tumours have the worst prognosis^13^, and have faster rates of metastases compared to HER2 + patients^14,15^. However, following primary tumour treatment, and a period of apparent dormancy (in some cases lasting > 10 years), ER + tumours frequently metastasise to the brain^16–18^. Therefore, identifying genomic alterations that occur uniquely in primary tumours that eventually metastasise to specific organs could help clinical management of the disease^18^.

We have previously used a candidate-gene approach to identify epigenetically dysregulated genes in BBM^19^. Following from this study, we wished to carry out a non-selective, genome-wide analysis to identify novel epigenetic changes that are common in breast tumours that metastasis to the brain. To identify candidate genes that are differentuialy methylated when comparing primary breast tumours and BBM we have carried out 450 K-methylation array analysis of BBM tumours and compared the genome-wide methylation status of these tumours to primary breast tumours from the Cancer Genome Atlas (TCGA). We then determined the methylation status of candidate genes in BBM and their originating primary breast tumour. This analysis has led to the identification of DNA methylation alterations that commonly occur in primary breast tumours that eventualy metastasis to the brain. We have also identified epigenetic changes that occur only after the tumor cells have disseminated from the primary breast tumours. In addition, as a first step towards the development of a non-invasive prognostic tool, we have validated the methylation status of the genes identified in patients’ serum. The identified epigenetic alterations may be used as potential non-invasive markers and new therapeutic targets for BBM patients.

## Materials and methods

### Patients and samples

Thirty fresh-frozen metastatic brain tumours (BBM) that originated from primary breast tumours (BP) were provided by The Walton Research Tissue Bank (WRTB), Liverpool and Brain Tumour North West (BTNW) Tissue Bank, Preston; BBM tumours were labelled BBM1 to BBM30. Formalin fixed paraffin embedded (FFPE) originating primary tumours (BP) from individual patients corresponding to their brain metastases (matched-pairs) were available for 11 of these tumours (BBM1, BBM2, BBM5, BBM7, BBM8, BBM10, BBM11, BBM12, BBM13, BBM14 and BBM15). These primary and BBM pairs were labelled as individual patients such as patients 1, patient 2, patients 5 etc. Patients’ serum was available for BBM1, BBM2, BBM5, BBM6, BBM7, BBM8, BBM10, BBM11, BBM12, BBM13. Serum was collected at the time of BBM Surgery. Receptor status information is available for 9 of the 11 primary tumour pairs, six of these are ER + ve, one is triple negative, one is ER/PR-ve, and one is HER2 + ve (with ER/PR unknown status). Additional clinical information is available through our previous publication^19^. The time between primary tumour surgery and removal of the brain metastasis ranges from 2 to 10 years.

An independent cohort of 40 primary breast tumours (BP) analysed during this study were ductal carcinomas; their clinical characteristics were described previously^19^. Molecular characterization was available for 20 of these tumours, 15 of these are ER + ve and three are triple negative. No brain metastases were observed in any of these patients. Seventeen of these patients had been screened for metastasis for ≥ 5 years from the time of primary tumour surgery and nine had been screened for ≥ 10 years.

The research ethics committee (North Wales REC: 11/WNo03/2) approved tissues from the research banks and informed consent was obtained from each patient. The project was carried out following local ethical approval (University of Wolverhampton Life Sciences Ethics Committee: LSEC/201,011/43). This study was conducted according to the principles expressed in the Declaration of Helsinki.

### Genomic DNA/RNA extraction

Genomic DNA was extracted from fresh-frozen BBM tumours using The *DNA isolation kit from cells and tissues* (Roche, Germany) as previously described^19^. Briefly, 25 mg of tissue was homogenised using lysis buffer and incubated at 37 °C for 30 min followed by the addition of Proteinase K and RNase solution. The samples were then centrifuged and processed according to manufacturer’s instructions. For FFPE samples, a *FFPE DNA extraction kit* (Qiagen, USA) was used as previously described^19^. Briefly, a small block of samples embedded with paraffin was cut into thin sections and mixed with xylene followed by 100% ethanol. The samples were then processed according to manufacturer’s instructions. The tumour-free circulating DNA from the patients’ serum was extracted using *ZR serum DNA kit* (Zymo research, USA). Briefly, 2 ml plasma from each patient was transferred to a conical shaped 50 ml universal tube. 8 ml of genomic lysis buffer and 10 μl of zymoBeads were added to each sample and placed in a shaker for two hours at room temperature. The samples were then processed further according to manufacturer’s instructions. Similarly, the total RNA was extracted using *EZ-RNA extraction kit* (Biological Industries, Israel). Briefly, fresh-frozen tumours were homogenized using lysis buffer followed by addition of extraction solution. The samples were then centrifuged and processed according to manufacturer’s instructions. The concentration of DNA and RNA was measured using *nanodrop2000* (Thermo Scientific, USA).

### Illumina BeadChip 450 K HumanMethylation array

Of 30 samples received, twenty-three BBM samples were used to assess the genome-wide methylation profilings of > 485 K individual CpG loci using Illumina BeadChip 450 K HumanMethylation Array (450 K array). The 450 K array profiling was carried out at Cambridge Genomic Services (CGS), UK. Chip processing was carried out based on 450 K array design according to manufacturer’s instructions. Signal intensities generated by Illumina *GenomeStudio* were converted to β-values and *BeadStudio* software was used to remove biases between the Infinium I and II probes. In order to remove the technical biases between the 450 K array downloaded from the TCGA (14 normal breast tissues and 14 primary breast tumours) and our array on 23 BBM, further normalisation was carried out using R statistical package^20^. Processing of array data was performed with the R packaged RnBeads (version 0.99.10) and normalized using the SWAN normalization option. The hg19 genome annotations were used during the analysis and sex chromosome data was excluded^21,22^. Data in figures and tables are derived from gene–gene comparisons using standard analysis options in the package. The tumour barcode for the tumour data downloaded from TCGA is given in Supplementary Fig. 1.

### Initial screening of candidate genes signatures hypermethylated or hypomethylated in brain metastases compared to primary breast tumours and normal breast tissues

In order to generate an initial candidate list of genes that are either hypomethylated or hypermethylated in BBM, we compared CpG methylation between normal breast tissues (BN), primary breast tumour tissues (BP) and breast to brain metastases (BBM) samples. For this, we downloaded 450 K methylation array data from The Cancer Genome Atlas (TCGA) for 14 normal breast tissues and 14 primary breast tumours (Supplementary Fig. 1A, B. Our analysis aimed to identify individual CpGs that gained methylation (hypermethylated) in BBM compared to primary breast tumour and normal breast tissue samples (see Supplementary Fig. 1C for an example). We also looked for individual CpGs that have lost methylation (hypomethylated) in BBM compared to primary breast tumors and normal breast tissues. (See supplementary Fig. 1D for an example). In order to generate an initial list of CpGs, that have either gained or lost methylation in BBM, we retrieved individual CpGs (array probes) with a β-value of ≥ 0.4 in 50% of the BBM tumours and < 0.4 in 50% of the primary tumours and normal tissue; previous studies have shown that β-values of ≥ 0.4 are associated with silencing of genes or significant loss of expression^23–26^. From this list of methylated CpGs we retained only those CpGs that had a diference in β-value of ≥ 0.15 between the metastatic and primary tumour sets. Similarly, In order to identify candidate hypomethylated loci in BBM, we selected CpG probes that had β-values < 0.4 in BBM and β-values of ≥ 0.4 in normal breast tissues and primary tumour tissues in at least 50% of each sample sets and then retained the CpG loci that have an average β-value differences of ≥ 0.15 between BBM and, normal breast tissues and primary breast tumours. The CpG loci that met these criteria and were significnaly differentialy methylated (P ≤ 0.05) across the different tissue types (Supplementary Table 1A) were carried forward for further analysis.

### Bisulfite conversion of DNA

Bisulfite conversion of genomic DNA from tumours was carried out using the *EZ DNA methylation kit* (Zymo Research Corp., USA). Briefly, 300 ng of genomic DNA was mixed with bisulphite conversion reagent and was incubated for 16 h at 50 °C. The samples were then processed according to manufacturer’s instructions. Bisulphite conversion of tumour-free circulating DNA (tfcDNA) from patients’ serum was carried out using *Epitect Bisulfite kit* (Qiagen, USA). Briefly, 200 ng DNA from the plasma was added to 200 μl sterile PCR tube and was mixed up with 85 μl bisulphite conversion reagent (bisulphite mix), 85 μl DNA protect buffer and RNase free water making up total volume of 140 μl. The samples were then processed according to manufacture’s instructions. Fully methylated, positive controls were generated by incubating genomic DNA with DNA methyltransferase, in the presence of S-Adenosyl methionine (SAM) (New England bio lab, USA) for 2 h at 37 °C prior to bisulfite conversion.

### Experimental validation of methylation status of individual genes

The methylation status of each gene corresponding to differentially methylated probes was determined by Combined Bisulphite and Restriction Analyses (CoBRA). Semi-nested PCR was carried out and DNA methylation status of candidate genes was determined by digesting CoBRA PCR products with BstUI or TaqI restriction enzymes (Fermentas, UK).

CoBRA primers were designed based on the standard primary designing criteria used in analyzing bisulphite converted DNA^27–29^. The CoBRA primers were designed in such a way that the region analysed included the specific CpG identified by the 450 K array and additional local CpGs to enable the reliable determination methylation status by restriction digestion (Supplementary Table 2A). The methylation status of genes in patients’ serum was determined using Methylation Specific PCR (Supplementary Table 2B).

### Experimental validation of expression of selected genes

Expression analysis of candidate genes was carried out using quantitative-reverse transcription PCR (qRT PCR). Due to the limited size of the tumor biopsies available, RNA was available only for BBM13, BBM15, BBM16, BBM20 and BBM30. Prior to the PCR, cDNA was prepared using QuanTect reverse transcription kit (QIAGEN, USA). β-actin gene was used as internal control for mRNA expression. The Ct value obtained were converted to the relative quantity of targets genes normalized with respect to internal control, and relative to a control sample. Due to the lack of normal tissue control samples (associated normal brest tissue was avalble only as FFPE), a BBM samples with median level of of expression for each qRTPCR among the samples set, was used as a control sample to calculate fold enrichment of other samples.

### Statistical analysis

Initial statistical tests to generate candidate CpG loci was carried out using the statistical package available in R^21^. During the experimental validation, only the samples where methylation differences were statistically significant between primary breast tumours and BBM were taken further. Fisher’s exact test was used to determine the statistical significance of methylation between BP versus BBM samples. P ≤ 0.05 was considered statistically significant.

### Patient survival analyses

Survival analyses of breast cancer patients were carried out to investigate the correlation of expression of candidate genes with clinical prognosis using online KM-plotter tools^30^. KM-plotter is an online tool capable of measuring the prognostic indication of 22,277 genes based on gene expression data and survival information of 6234 breast cancer patients (Affymetrix HGU133A and HGU133 microarrays datasets) downloaded from Gene Expression Omnibus (GEO)^31^. This tool segregates patients on high or low expressing groups based on median or a lower/upper quartile as a cut off to measure a statistical significance of gene expression with patient survival. Logrank P < 0.05 was considered statistically significant.

## Results

### Identification of differentially methylated genes in BBM

**Figure 1:**
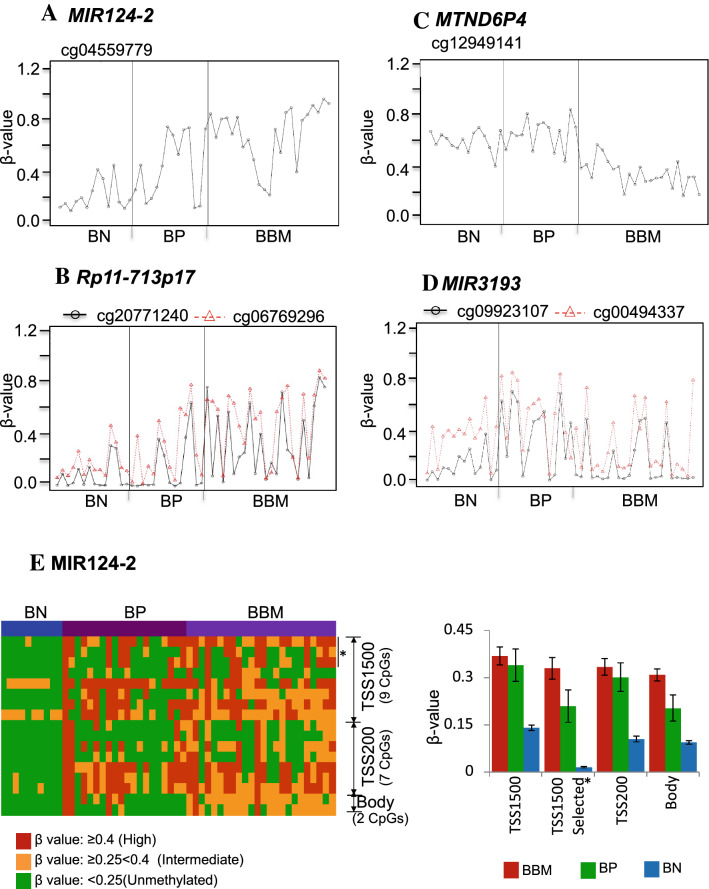
Genome-wide DNA methylation analyses by 450 K array for BBM, primary breast tumours (BP) and normal breast tissues (BN) identified CpG loci which have either gained methylation (hypermethylated) or have lost methylatioin (hypomethylated) in BBM compared to BP and BN tumours. Representative examples of gene-associated CpG loci that have gained methylation (hypermethylated) in BBM (**A,B**) or have lost methylation (hypomethylated) in BBM (**C,D**) compared to BP and BN (as measured by β-value). (**E**) Regional methylation pattern of *MIR124-2* showing the the location of all CpG loci present on the 450 K array relative to the transcription start of the gene and the differing methylation levels for each loci in BBM, BP and BN. * denotes the location of the differentially methylated CpG that was identified by the bioinformatic analysis of 450 K-array data from BN, BP and BBM.

We identified differentialy methylated CpGs between breast-to-brain metastases (BBM) and non-metastatic primary breast tumours (BP) and normal breast tissue (BN) by comparing Illumina BeadChip 450 K methylation data that we generated for 23 BBM to data for normal breast tissues and primary tumours from TCGA. First, an initial screening of genes based on β-value (see method section for details) was carried out to identify CpG loci that were either hypermethylated (Fig. 1A,B) or hypomethylatled in BBM (Fig. 1C,D) compared to normal breast tissues and primary breast tumours. This identified 26 candidate BBM hypermethylayted loci (14 genes) and 58 BBM hypomethyaled loci (40 genes) (p ≤ 0.05; Supplementary Table 1A). In order to concentrate on loci consisting of the most frequently occurring and largest β-value differences between the two tumour types, we further generated a candidate list that contained loci that are either hypermethylated in BBM (β-value > 0.4) in ≥ 50% of tumours analysed or hypomethylated in BBM ((β-value < 0.4) in ≥ 50% of tumours analysed compared to BP (See Fig. 1A–D and Supplementary Fig. 2 for representative data of genes that matched these criteria). This shortlisting identified 6 hypermethylated genes that are frequently methylated in BBM samples compared to BN and BP and 9 hypomethylated genes that are infrequently methylateld in BBM compapred to BN and BP samples (Supplementary table 1B).

In addition, we examined the methylation level of all CpG loci available based on the 450 K array design that are associated with our candidate genes which we refer to as the regional methylation level of each gene. The 450 K array design provides annotations for each CpG includin genomic location of the CpGs in the array. We retrieved all CpGs corresponding to our candidate genes and outlined where each of the CpG sites are located in relation to the gene structure: TSS1500, (1500 nucleotides upstreams of transcription start site), TSS200 (200 nucleotides upstream of transcription start site), 1^st^ Exon, or body regions (downstream of the TSS or the first Exon) individually for all samples in relation to the gene structure (Fig. 1E, supplementary Fig. 3, and supplementary Fig. 9 Left panel). We determined an average methylation level of each CpGs in each sample type *i.e.* BN, BP and BBM in individual structural regions of each gene (Fig. 1E, supplementary Fig. 3, supplementary Fig. 9; right panel). Regional methylation maps have been constructed only for those candidate genes where there is sufficient 450 K probe density to generate informative figures.

### *RP11-713P17.4*, *MIR124*-2, *NUS1P3* are hypermethylated in brain metastases compared to primary breast tumours

All candidate genes were validated for their methylation status using Combined Bisulpite and Restriction Analysis (CoBRA), this is a robust and reliable method that is not prone to false positive results^32^. Primers were designed to amplify the probe regions identified in the 450 K arrays using standard criteria published previously^19,23^.

**Figure 2:**
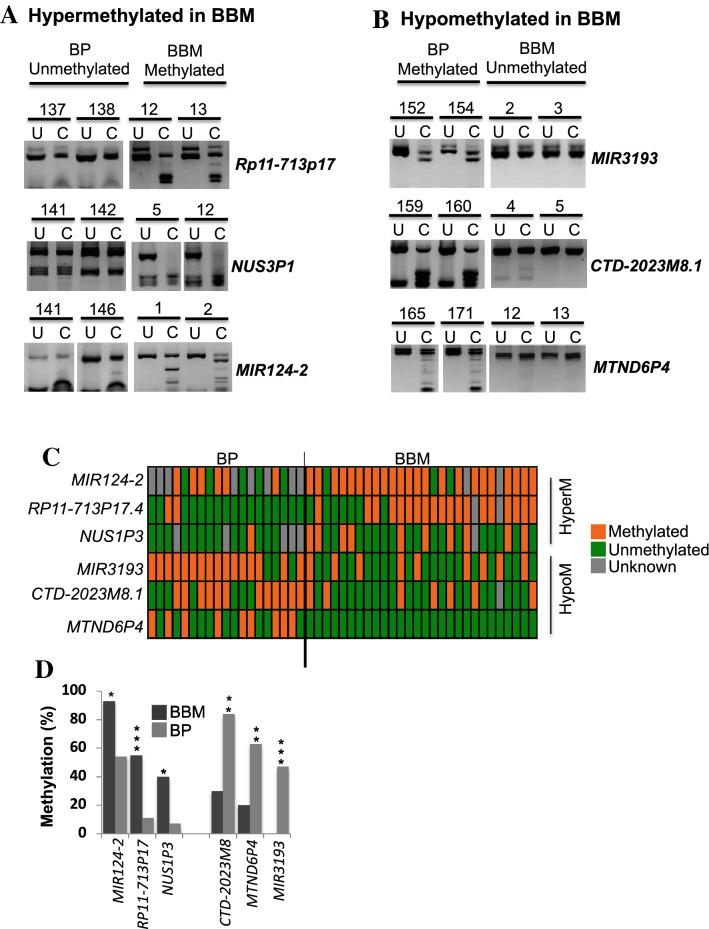
Representative examples of experimental validation of methylation status of candidate genes hypermethylated in breast to brain metastases (BBM) in a cohort of non-metastatic primary breast tumours (BP) (n = 20) and BBM (n = 30) using Combined Bisulphite and Restriction Analyses (CoBRA). (**A**) The two genes *RP11*-713P17.4 and *NUS1P3* are infrequently methylated in BP and frequently methylated in BBM (In addition, *MIR124-2* methylation is enriched in BBM compared to primary breast tumours). In contrast, (**B**) *MIR3193, CTD-2023M8,* and *MTND6P* are frequently methylated in BP and infrequently methylated (hypomethylated) in BBM. (**C**) An overview of the methylation status of genes that are hypermethylated or hypomethylated in BBM in all BBM and BP samples analysed by CoBRA (each box represents a tumour). (**D**) Summary of methylation status of genes in primary and metastatic brain tumours, *: Indicates statistical significant difference between primary and BBM tumours. BBM: Breast to Brain Metastases, BP: Primary breast tumours, U: Uncut/control sample, C: cut by restriction enzyme, , HyperM: hypermethylated in BBM, HypoM: Hypomethylated in BBM.

First, we validated the six shortlisted candidate hypermethylated genes (see supplementary table 1B), by CoBRA in 30 BBM, that included the 23 BBM samples used in the methylation array. Then, using the CoBRA method, the methylation level of these genes was examined in an independent cohort of 20 primary breast tumours (BP) with no evidence of metastatic progression. Comparison of the results from these two tumour groups allowed us to determine if the methylation of these genes is more commonly found in the brain metastases than in BP. Of the 6 candidate genes, 2 were infrequently methylated in BP tumours; *RP11-713P17.4* and *NUS1P3* (Fig. 2A,D and Supplementary Fig. 4B, S4C, S10A-C). These genes were found to be methylated in 10% and 7% of primary tumours but 73% and 55% of BBM respectively. While *MIR124-2* was methylated in 55% of the primary tumours and 88% of BBM (Fig. 2A–D, Supplementary Fig. 4A). This suggests that *RP11-713P17.4* and *NUS1P3* are frequently methylated in BBM but not in BP, and *MIR124-2* methylation frequency is enriched BBM compared to primary breast tumours (Fig. 2A–D and Supplementary Fig. 4A–C, Supplementary Table 1C). The methylation status of genes validated by CoBRA that were not differentially methylated are presenterd in Supplementary Fig. 6.

### *MIR3193*, *CTD*-*2023M8*.1 and *MTND6P4* are hypomethylated in brain metastases compared to primary breast tumours

Our methylation array analysis identified a panel of nine candidate genes that were hypometylated in BBM compared to BP and BN (see supplementary table 1B). As above, these genes were also validated using CoBRA in 30 BBM that included 23 BBM samples used in the methylation array and in an independent cohort of 20 primary breast tumours. This CoBRA analysis identifed three genes that were frequently methylated in BP and infrequently methylated in BBM tumours; *CTD-2023M8.1, MIR3193* and *MTND6P4* were methylated in 26%, 29% and 0% of BBM samples respectively (n = 30) (Fig. 2B–D, and Supplementary Fig. 4D–F, S11A-C) and were methylated in 63%, 67% and 47% non-metastatic BP respectively (n = 20) (Fig. 2B–D Supplementary Fig. 4D-F).

In summary, experimental validation of gene methylation status in BBM and primary breast tumours has identified six genes that are differentially methylated in BBM compared to BP (Fig. 2C,D). Of these, the promoter regions of three genes (*MIR124-2*, *RP11-713P17.4*, and *NUS1P3*) were hypermethylated in BBM and three genes (*MIR3193*, *CTD-2023M8.1* and *MTND6P4*) were hypomethylated in BBM relative to the primary tumours (Fig. 2C,D). As was seen following the initial 450 K array analysis, the differences in the methylation status between BBM and BP for these genes analysed by CoBRA were statistically significant *i.e. MIR124*-2 (p = 0.03), *RP11-713P17.4* (p = 0.0001), *NUS1P3* (p = 0.004), *MIR3193* (p = 0.0003), *CTD-2023M8*.1 (p = 0.01) and *MTND6P4* (P = 0.0001) (Fig. 2D, Supplementary Table 1C).

### Methylation status of candidate genes in BBM samples and their originating primary breast tumours

To determine if the common BBM-associated methylation events are also detectable in the primary tumours that the metastases are deriverd from, the methylation status of these genes was determined in BP and associated secondary BBM tumours from 11 individuals.

Primary tumour material was available in the form of FFPE sections. We could bisulphite convert, amplify and analyse the promoter regions for *RP11-713P14.4, MIR3193*, *MTND6P4* and *CTD-2023M8.1* in these corresponding primary tumours. However, we were unable to amplify some regions in all 11 samples and *MIR124-2* and *NUS1P3* promoter regions were refracrtive to amplification in all FFPE primary samples.

**Figure 3:**
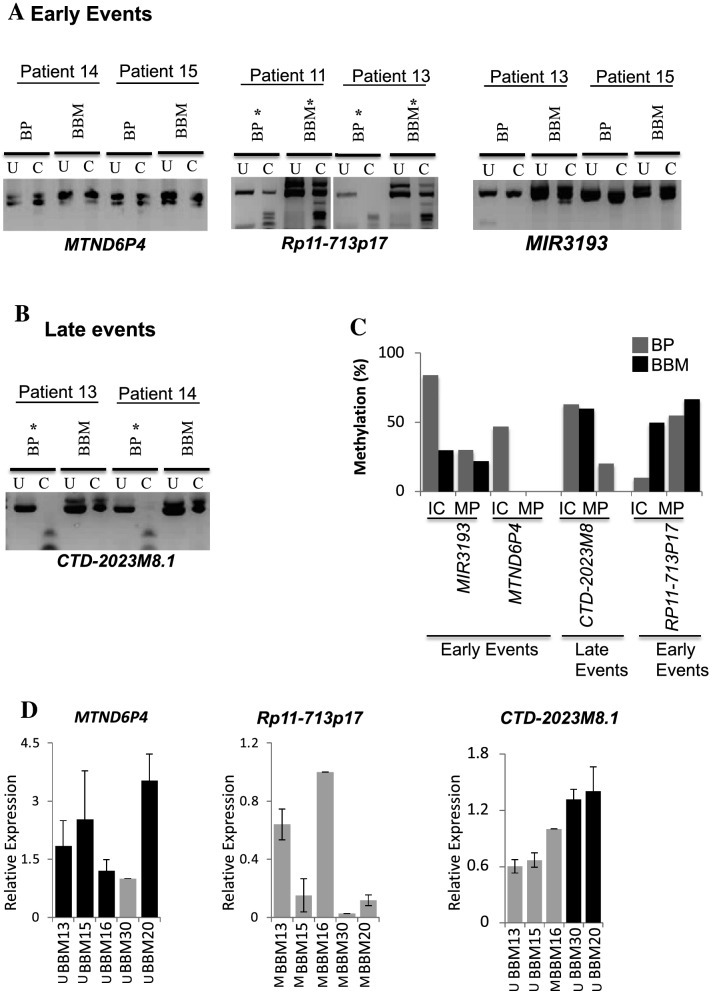
Methylation status of BBM-Hypermethylated and BBM-Hypomethylated genes in BBM and their originating BP tumours from individual patients. (**A**)** Early events**; Methylation of *MTND6P4* (left panel), *RP11-713P14.4* (middle panel) and *MIR3193* (right panel) in originating BP is the same as found in their corresponding BBM in individual patients (**B**)** Late events**; Methylation status of *CTD-2023M8.1* in orginating BP is different to their corresponding BBM tumours (loss of methylation at this region occurs in the metastasis). (**C**) Summary of methylation status of genes denoting early events and late events in individual patients/matched pairs (MP) and an independent cohort (IC) of primary breast tumours. (**D**) Expression analyses of genes by quantative reverse transcriptase PCR (qRT-PCR) in representative BBM tumours showing methylation-expression correlation, tumours with genes that are methylated (M) have a low level of expression. BP: Breast Primary tumour, BBM: Brain Metastases, U: Uncut/Control sample, C: cut by methylation specific restriction enzyme, *: Methylated samples, IC: independent cohort, MP: matched pairs from individual patients.

*MTND6P4* and *MIR3193* are frequrently methylated in primary breast tumours tumurs (9/19, 16/19; see Fig. 2). However, we found that these regions are commonly unmethylated in metastasis-originating primary tumours (0/5, 3/9) and their corresponding BBM tumours (Fig. 3A; left panel, 3C and Supplementary Fig. 5A,C). Similarly*, RP11-713P17.4* is infrequently methylated in primary breast tumours (non-metasatic; 2/19 see, Fig. 2). However, in the primary tumours that proceed to metastasise to the brain this region is found to be methylated (3/6) (Fig. 3A; right panel, 3C, Supplementary Fig. 5B). These results suggest that the differential methylayion we observed between unrelated primary tumours an BBM may be a result of differences that occur early during the developmet of the tumours that metastasize as they are common to the originating primary tumour and the associated metastatic tumour. From hearin we will refere to these as early events (in metastatic tumour evolution).

*CTD-2023M8.1* is frequently methylated in primary tumours with no history of distant metastasis (12/19) but infrequently methylated in BBM (7/27) (see Fig. 2). *CTD-2023M8.1* was also found to be methylated in metastasis-originating primary tumours However, *CTD-2023M8.1* was not methylatyed in the corresponding BBM. This Suggests that this genomic change (the loss of methylation) was selected for after the metastasising tumour cells had left the primary tumour (Fig. 3B,C, Supplementary Fig. 5D). From hearin we will refere to this as a late event (in metastatic tumour evolution).

In addition, we carried out quantitative reverse transcription PCR (qRT PCR) to determine the expression status of these genes in the same cohort of BBM tumours that was used for 450 K methylation and experimental validation of methylation status (Fig. 3D, Supplementary Fig. 7). The expression was normalized against β-actin. As there was no RNA available from the primary tumors the fold change was determined relative to the expression level of a median ΔCT value for each transcript analysed. We have found that the genes that have promoter methylation have relatively low RNA levels (relative expression < 1) in those tumours. Similarly, the genes which are unmethylated, are expressed in those tumours (relative expression > 1). We have found that some of the samples/ genes that are not methylation have also low level expression which could be attributed to genomic changes other than the DNA methylation (Fig. 3D, Supplementary Fig. 7).

### Examination of methylation status of candidate genes in tumour free circulating (tfc) DNA

**Figure 4:**
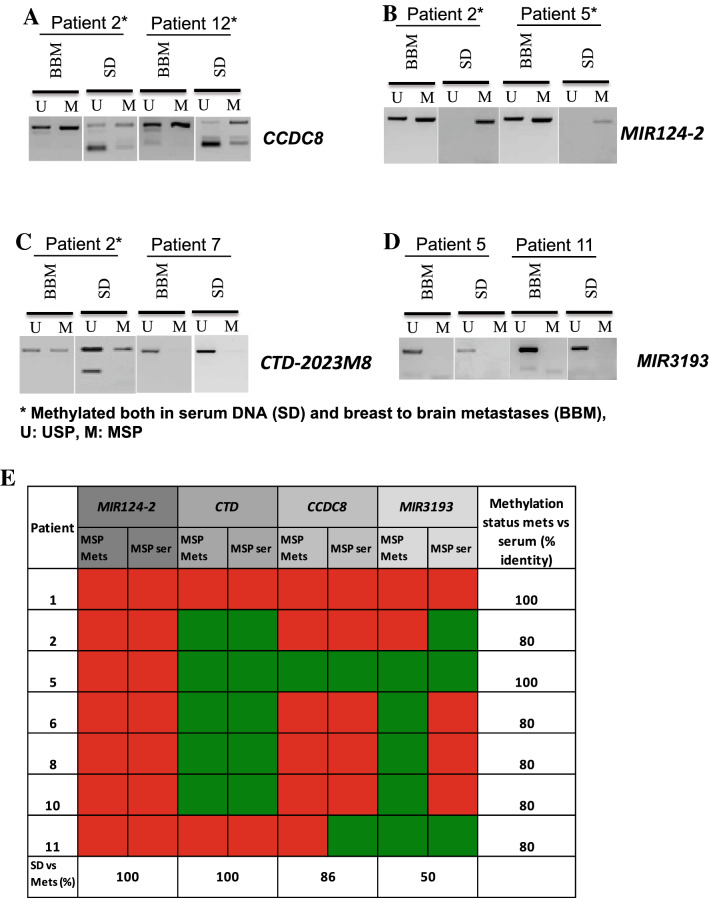
Methylation status of candidate genes in BBM, and tumour free circulationg DNA (tfcDNA) in patients’ serum determined by Methylation Specific PCR (MSP). (**A,B**) Methylation status of candidate genes denoting early events (*CCDC8* and *MIR124-2*) and (**C,D**) late events (*CTD-2023M8* and *MIR3193*) in BBM and their corresponding tumour free circulating DNA in individual patients (presence of a PCR product in the MSP lane is indicative of methylation in the region analysed). (**E**) Summary of methylation status of BBM and serum DNA in individual patients; Red suqares indicate methylation identified, Green squares indicate no methylation identified. BBM: Brain Metastases, SD: Serum DNA, U: Unmethylation Specific PCR; USP, M: Methylation Specific PCR; MSP, *: Methylated samples.

In order to investigate if the methylation status of candidate genes in BBM is similar to their methylation status in circulating DNA isolated from patients’ serum (at the time of metastasis surgery), we determined the methylation status of *MIR124*-2, CTD-*2023M8* and *MIR3193* and *CCDC8* in patients’ serum (*CCDC8* had previously been identified as methylated in BBM in our earlier study^19^) by methylation-specific PCR (MSP). Serum was available from those patients whose originating primary tumours and BBM were also available. The methylation status of tfc DNA isolated from serum at *MIR1242*, *CTD-2028M8*, *CCDC8* and *MIR3193* was the same as that seen in BBM in 100%, 100%, 83% and 50% of the samples respectively (Fig. 4A–E, Supplementary Figs. 8, 12). It is important to note that while we belive this analysis a useful in showing that these epigenetic marks can be identified in tfc DNA, this analysis is limited as matched blood samples to the retrospectively collected primary breast tumours were not available.

### Expression status of *MIR124-2* gene correlates to the clinical prognosis of patients

We wished to carry out survival analyses of breast cancer patients to investigate the correlation of expression of candidate genes with clinical prognosis using KM-plotter tools using data from the Gene Expression Omnibus (GEO)^31^. However, prognosis data was not available for *mir3193* and *NUS1P3* or the log-non-coding RNAs that we have identified. Data was avalalber for *MIR124-2* and, in addition, we also performed KM analysis for three genese we have previously found to be frequently methylated in BBM (*BNC1*, *CCDC8* and *GALNT9)*^19^.

**Figure 5:**
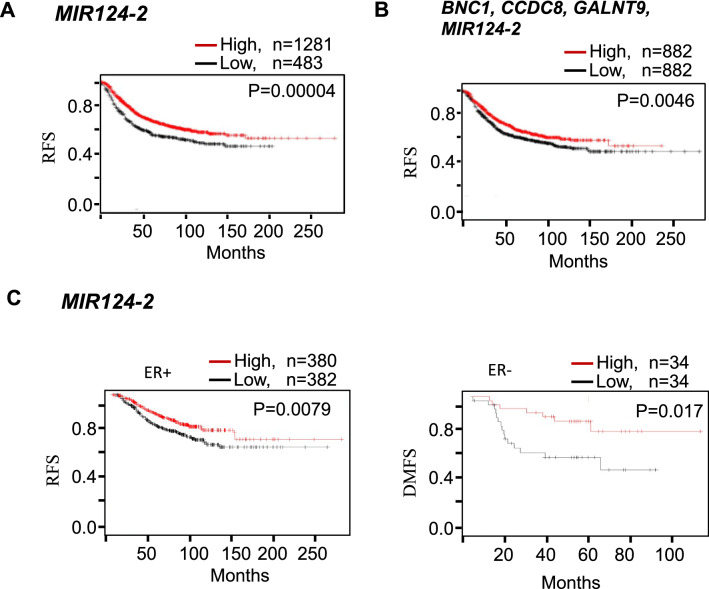
Loss of expression of BBM-hypermethylated genes (candidate metastasis suppressor genes) correlates with survival in breast cancer patients. (**A**) Kaplan–Meier analysis shows that low expression of *MIR124-2* correlates to poor relapse free survival (RFS) of breast cancer patients (p = 0.00004). (**B**) Kaplan–Meier analysis using a combined four-metastasis suppressor gene signature (*BNC1*, *CCDC8*, *GALNT9* and *MIR124-2*) shows that loss of expression of these genes in combination correlates to poor RFS of breast cancer patients (*p* = 0.0046). (C) Low expression of MIR124-2 is associated with poor prognosis of ER + and ER- breast cancer patients (p = 0.007 and 0.017 respectively).

Kaplan–Meier analyses showed that low expression of *MIR124-2* (that we have shown here to be frequently hypermethyated in BBM) correlates to poorer clinical outcomes (Fig. 5). We have found that the high expression of *MIR124-2* correlates to better relapse-free survival (RFS) of breast cancer patients (p = 0.00004 (Fig. 5A). Furthermore, we carried out combined prognosis of the four candidate genes (*BNC1*, *CCDC8*, *GALNT9*, *MIR124-2)*, three of which (*BNC1*, *CCDC8*, *GALNT9*) were reported to have metastatic suppressive functions in our previous study^19^. This combined analysis shows that higher combined expression of these 4 genes together correlated to better RFS (P = 0.0046) (Fig. 5B). In addition, we carried out survival analyses of ER + and ER- patients separately to investigate if expression of our candidate genes is associated with ER receptor expression in breast cancer patients; the data indicates that the expression of *MIR124* is independent of estrogen receptor (ER + or ER-) status in breast cancer patients (Fig. 5C).

## Discussion

The aim of this study was to identify genes that are frequently epigeneticly dysregulated in breast to brain metastases (BBM) using a genome-wide approache. Our study identified 6 genes (*RP11-713P17.4*, *MIR124*-2, *NUS1P3, MIR3193*, *MTND6P4* and *CTD-2023M8.1*) of which, *RP11-713P17.4*, *MIR124*-2 and *NUS1P3* were frequently hypermethylated in BBM whereas *MIR3193*, *MTND6P4* and *CTD-2023M8.1* were frequently hypomethylated BBM compared to BP and BN. Notably, all of these genes identified were non-protein coding genes; two microRNAs (*MIR3193* and *MIR124-2*), two long intergenic non-coding RNA (lincRNA) genes (*RP11-713P17.4* and *CTD-2023M8.1*) and two pseudogenes (*MTND6P4* and *NUS1P3*).

Recently dysregulated lncRNAs have been shown to be associated with various cancer types^33,34^, EMTs and metastases^35^. There is growing evidence that non-coding RNA could be a class of novel biomarkers or therapeutic targets in multiple cancers^36,37^ and prevous studies have reported that non-coding RNAs are epigenetically dysregulated in cancer^38,39^. This study has identified non-coding RNAs that are dysregulated by DNA methylation that, potentially, could be used as a DNA methylation prognostic markes in BBM.

The methylation level of non-protein coding genes *MIR124-2, NUS1P3* and *RP11-713P17.4* was enriched in BBM tumours compared to primary breast tumours. Previous studies have reported that *MIR124* is associated with inhibition of invasion and metastases of breast and lung cancers^40^, oral squamous cell carcinoma^41^ and pancreatic adenocarcinoma^42^. *MIR124* is epigenetically dysregulated in hepatocellular carcinoma (HCC) cell lines^43^ and its silencing is also associated with poor clinical prognosis of colorectal carcinoma^44^. Recently, the methylation status of *MIR124-2* has been proposed as a prognostic marker associated with cervical cancer in human papoloma Virus (HPV) positive women^45,46^. *MIR124-2* is one of three independent precursors genes; *MIR124-1*, *MIR124-2*, and *MIR124-3*, which are processed to form *MIR124*. Interestingly, the other precursors *MIR124-1* and *MIR124-3* are also methylated in BBM compared to BP and BN (Supplementary Fig. 3) suggesting that *MIR124* is dysregulated in BBM. Recent studies have reported that *MIR124-2* suppresses proliferation, aggressiveness, apoptosis, and invasion of osteosarcoma^47,48^. *MIR124* is abundantly expressed in the nervous system where it contributes to regulation of alternative splicing and plays a crucial role in the differentiation of progenitor neuronal cells^49,50^. *MIR124* also contributes to glial cells quiescence and is involved in repression of migration and invasion of various cancers through its targets^49^.

*NUS1P3* is a processed pseudogene of its parental gene *NUS1(Dehydrodolichyl Diphosphate Synthase Subunit). NUS1*, also known as *NOGO-B Receptor* is expressed in most tissues^44,51,52^. *NUS1* down regulates epithelial markers such as E-cadherin and increases mesenchymal markers contributing to EMT in cervical cancer promoting invasion and metastasis^53^. In addition, *NUS1* dysregulation is associated with various cancer types^54^ including ER/PR/HER2 positive breast tumours^55^. There is growing evidence that pseudogenes are dysregulated in cancer and this dysregulation may modulate thire intereaction with either parental genes or other gene loci regulating transcriptional, and post transcriptional activities^56,57^. In our study, *NUS1P3* is unmethylated in primary tumours suggesting its expression, which may lead to increased expression of *NUS1*. Furthermore, increased expression of *NUS1* could promote Epithelial to Mesenchymal Transition (EMT) in breast cancer contributing to invasion and metastases to the brain. It is possible that *NUS1P3* acts as a competitive endogenous RNA (ceRN)A for *NUS1* and the silencing of *NUS1P3* in metastases found in the brain lead to down-regulation of *NUS1* concorant with Mesenchymal to Epithelial Transition (MET). These findings are consistent with our finding that silencing of *MIR124-2* and *NUS1P3* through promoter methylation in BBM samples may provide a selective advantage for metastasised tumours to survive and to proliferate in the brain microenvironment.

*RP11-713P17.4* is a long intergenic non-coding RNA (lincRNA) gene. LincRNAs are gene-associated transcripts that are associated with open chromatin marks such as histone modification sites and epigenetic regulation of transcription, RNA stability, and recruitment of protein complexes^58–60^. LincRNA are associated with crucial biological functions such as cellular growth and differentiation, development, and apoptosis^59,60^. There is growing evidence of epigenetic reglations of lincRNAs in cancers^61,62^, and further mechanistic studies are required to investigate the molecular mechanistic role of *RP11-713P17.4* in BBM and cancer metastases.

Three non-protein coding genes *MIR3193, MTND6P4* and *CTD-2023M8.1* are hypomethylated in BBM compared to primary tumours and normal breast tissues. The microRNA, *MIR3193* was one of 209 novel micro RNAs identified by deep sequencing melanomas^63^. Furthermore, *MIR3193* is frequently upregulated in glioma compared to normal brain tissue^64^.

*MTND6P4* is a processed pseudogene of its parental gene *MTND6* (*ND6*) that codes for the protein Mitochondrially Encoded NADH Dehydrogenase 6 (MTND6). MTND6 provides a quinone binding sites and is one of the six subunits (*ND1-ND6*) of the complex I in electron transport chain (ETS) in mitochondria. Mutations in *MTND6* are associated with an increase in metastatic potential that was associated with low NADH and high reactive oxygen species (ROS) in lung and breast cancer cell lines^65,66^ and heptatocellular carcinoma^67^. Mutation in one of the subunits of ETS leads to low oxidative phosphorylation and increased glycolytic activity of mitochondria contributing to aggressiveness of childhood Acute Lymphoblastic Leukemia^68^. It is possible that epigenetic dysregulation of *MTND6P4* may contribute to an energy shift towards glycolysis leading to acidosis with microenvionment changes that provide powerful growth advantages and invasive potential to the tumour cell^69^. A principle emerging role of pseudogenes is to act as competitive endogenous RNAs (ceRNA), a sponge for molecules that interact with mRNA (such as miRNA) thus positively influencing the expression of their parental gene^70^. *MTND6P4* may regulate its parental gene *MTND6* and hypomethylation and subsequent overexpression of *MTND6P4* may lead to changes in oxidative phosphorylation activies, glycolysis and brain microenvironment contributing to growth advantages to tumours via upregulation of *MTND6*.

In our study, *MIR3193, MTND6P4* and *CTD-2023M8.1* were frequently methylated in a cohort of non-metastatic primary breast tumours and infrequently methylated in BBM. The two genes *MIR3193* and *MTND6P4* were commonly unmethylated in BBM and their originating primary breast tumours in individual patients suggesting that the demethylation/hypomethylation of *MIR3193* and *MTND6P4* is an early event during tumour evolution. It further suggests that *MIR3193* and *MNTND6P4* have metastatic promoter function, which are silenced in normal breast tissues and primary breast tumours due to methylation. Similarly, *CTD-2023M8.1* is frequently methylated in non-metastatic primary breast tumours. It is also frequently methylated in metastasis-originating primary tumours. However, it is frequently unmethylated in BBM. This suggests that the hypomethylation of *CTD-2023M8.1* in BBM is a late event that may occur only after the tumour cells have left the primary site.

*RP11-713P17.4* is infrequently methylated in primary breast tumours and normal breast tissues but frequently methylated in BBM and their originating primary tumours in individual patients suggesting that the promoter hypermethylation of *RP11-713P17.4* is an early event during BBM. The novel non-protein coding genes identified in this study may regulate invasion and metastasis directly or by regulating other protein coding genes. However, their regulatory functions and their target genes have not been reported before. Functional studies are required to determine the role of these genes in BBM.

In addition, our study shows that patients’ serum could be useful to detect the methylation status of tumour associated circulating DNA in BBM suggesting a potential method of prognostic analysis. For this, a panel of genes could possibly be developed as prognostic markers for BBM. However in this study, patients’ serum taken at the time of primary tumour diagniosis was not available to determine if the methylation status of these genes could be used as non-invasive prognostic markers.

Taken together, our study has identified a panel of six novel non-protein coding genes (miRNAs, pseudogenes and long intergenic/non-coding RNAs) of which *RP11-713P17.4*, *NUS1P3, MIR3193*, *MTND6P4* and *CTD-2023M8.1* have been reported for the first time as epigenetically dysregulated genes in cancer and in metastases. *MIR124-2* has previously been reported as an epigenetically dysregulated gene in cancer^43–46,71–74^, however, its role in breast cancer metasetases has not been reported previously. The non-coding RNA genes^75^ are a part of broad epigenetic network that are emerging as critical regulators in human diseases and cancers^76,77^. The genes that we have identified may be of use in the development of prognostic biomarkers and as therapeutic targets. Larger-scale studies will be required to determine the usefulness of these findings to translate to the clinic.

## Supplementary Information

Supplementary Information 1.

Supplementary Information 2.

Supplementary Information 3.
