# Supplementary material for: Genome-wide methylation analyses identifies Non-coding RNA genes dysregulated in breast tumours that metastasise to the brain
Source: Sci Rep. 2022 Jan 20;12:1102. doi: 10.1038/s41598-022-05050-z (PMC8776809; doi:10.1038/s41598-022-05050-z)
Supplement: Supplementary file 3 — Supplementary Information 3. [file 41598_2022_5050_MOESM3_ESM.pdf]

Supplementary Figure 1

A

Normal breast tissues

TCGA-AC-A23H-11A-12D-A161-05
TCGA-BH-A204-11A-53D-A161-05
TCGA-BH-A209-11A-42D-A161-05
TCGA-E9-A1RB-11A-33D-A161-05
TCGA-E2-A1LI-11A-23D-A161-05
TCGA-E2-A1LS-11A-32D-A161-05
TCGA-E9-A1RC-11A-33D-A161-05
TCGA-E9-A1RD-11A-33D-A161-05
TCGA-BH-A208-11A-51D-A161-05
TCGA-E9-A1RF-11A-32D-A161-05
TCGA-D8-A1X6-01A-11D-A14N-05
TCGA-D8-A1XJ-01A-11D-A14N-05
TCGA-AC-A23H-11A-12D-A161-05
TCGA-AC-A23H-11A-12D-A161-05

B

Primary breast tumours

TCGA-A2-A3XS-01A-11D-A230-05
TCGA-EW-A1P8-01A-11D-A145-05
TCGA-EW-A1P1-01A-31D-A14H-05
TCGA-AR-A2LH-01A-31D-A18O-05
TCGA-GM-A2D9-01A-11D-A18O-05
TCGA-GM-A2DA-01A-11D-A18O-05
TCGA-AC-A2FM-01A-11D-A19Z-05
TCGA-EW-A1P0-01A-11D-A145-05
TCGA-BH-A1FH-01A-12D-A13K-05
TCGA-A2-A0SW-01A-11D-A10P-05
TCGA-A2-A0T2-01A-11D-A10P-05
TCGA-AR-A0TZ-01A-12D-A10P-05
TCGA-AR-A0U2-01A-11D-A10A-05
TCGA-A2-A0SV-01A-11D-A10P-05

C

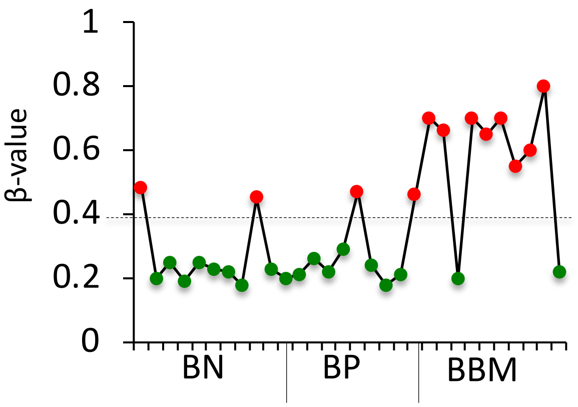

D

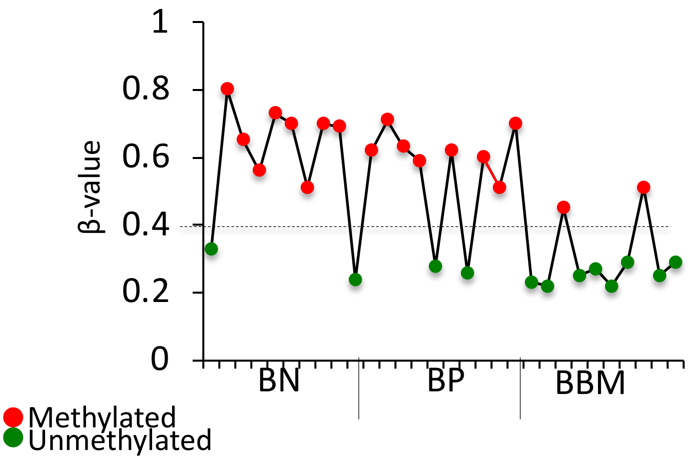

Supplementary Figure 1 A, B

BN and BP Tumour barcodes from TCGA used in this study

Supplementary Figure 1 C, D

Expected representative example of a single CpG probe β-values across normal (BN), Primary tumour (BP) and metastatic tumour (BBM) for a loci that has gained methylation (is hypermethylated) **(C),** or has lost methylation (is hypomethylated) **(D) in** BBM compared to BN and BP.

Supplementary Figure 2

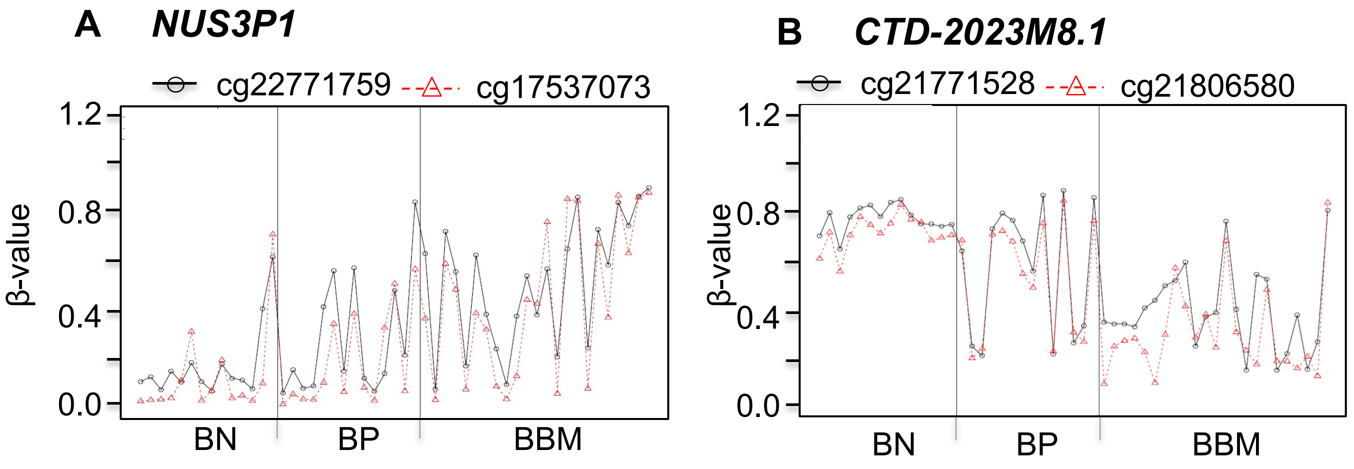

Supplementary Figure 2

DNA methylation analyses from our in-house 450K array for BBM and 450K data from TCGA for primary breast tumours (BP) and normal breast tissues (BN).

(A) CpG loci corresponding to *NUS1P3*, which is hypermethylated in BBM and **(B)** *CTD-2023M8* which is hypomethylated in BBM, compared to to BP and BN tumours.

Supplementary Figure 3

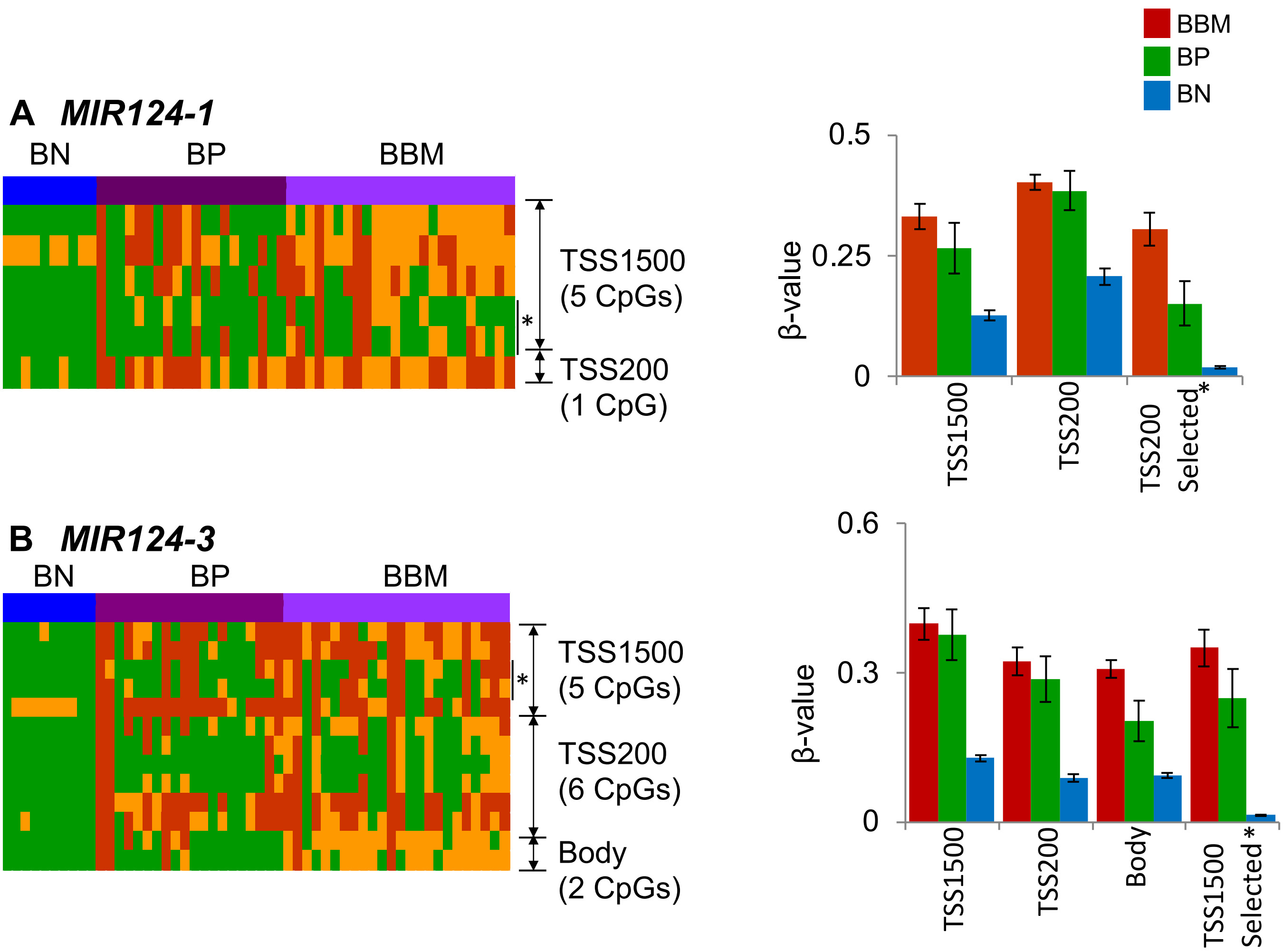

Supplementary Figure 3

Global methylation pattern of candidate genes

A) ***MIR124-1*** **and B)** ***MIR124-3***, showing the location of all CpG loci present on the 450K array relative to the transcription start of the gene and the differing methylation levels for each loci in BBM, BP and BN. This analysis shows an increase in methylation at multiple loci in BBM.

Supplementary Figure 4

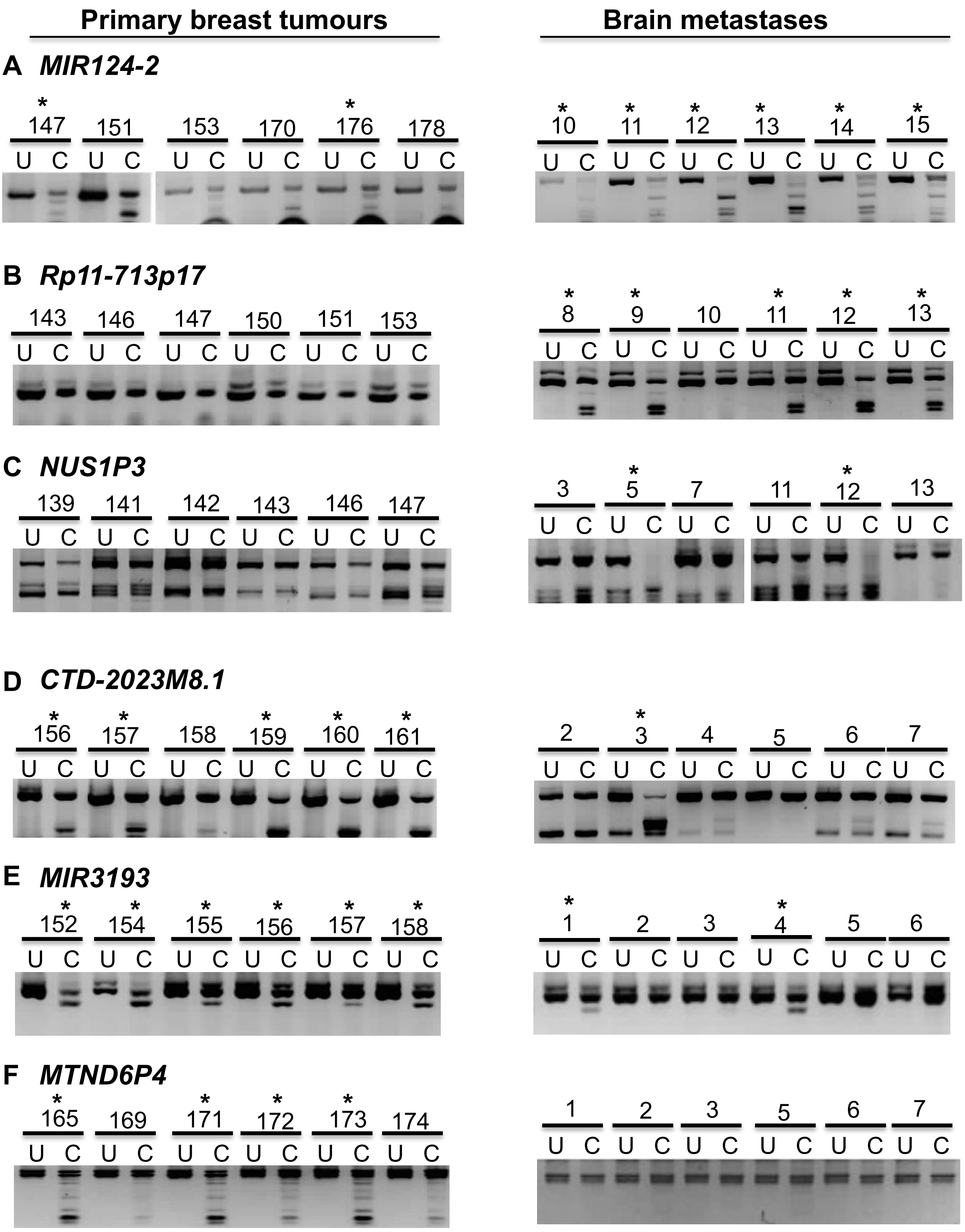

Supplementary Figure 4

Representative CoBRA images for methylation analyses of BP and BBM samples.

(A) Methylation status of *MIR124-2* is enriched in brain metastases. The two novel non-protein coding genes, **(B)** *RP11*-713P17.4 and **(C)** *NUS1P3* are infrequently methylated in BP and frequently methylated in BBM. In contrast, three other non-protein coding genes, **(D)** *CTD-2023M8.1* and **(E)** *MIR3193* are frequently methylated in BP and infrequently methylated in BBM. Similarly, a non-protein coding gene, **(F)** *MTND6P4* is frequently methylated in BP and not methylated in any of the BBM samples analysed. BBM: Breast to Brain Metastases, BP: Primary breast tumour, U: Uncut/control sample, C: cut by restriction enzyme, *: methylated samples

Supplementary Figure 5

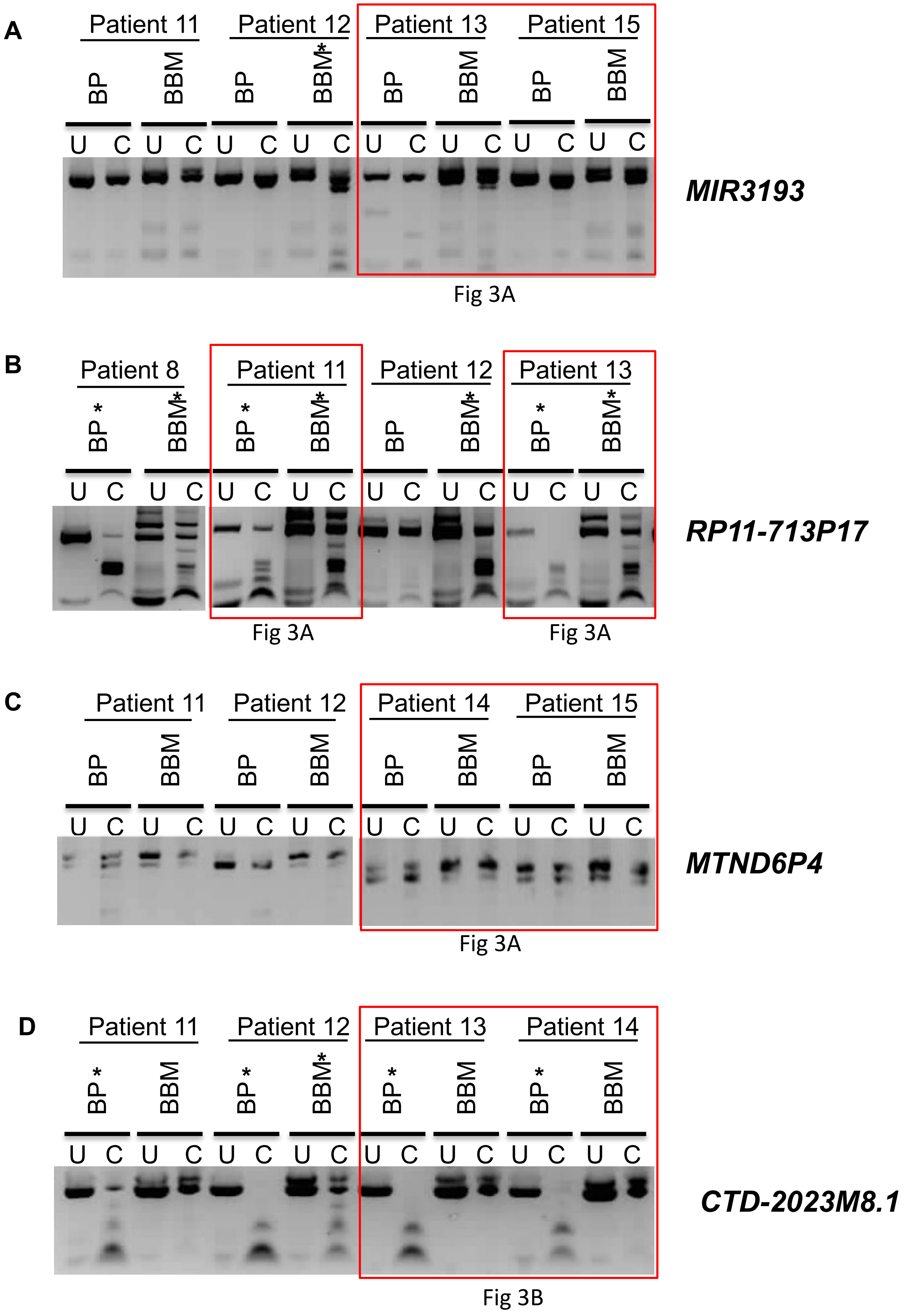

Supplementary Figure 5

Methylation status of candidate genes in BBM and their originating primary tumours (raw gel images).

(A) *MIR3193* is unmethylated both in BBM and its originating primary tumours from individual patients **(B)** *RP11-713P17.4* is methylated both in BBM and its originating primary tumours. **(C)** *MTND6P4* and **(D)** *CTD-2023M8.1* are unmethylated both in BBM and their originating primary tumours.

Supplementary Figure 6

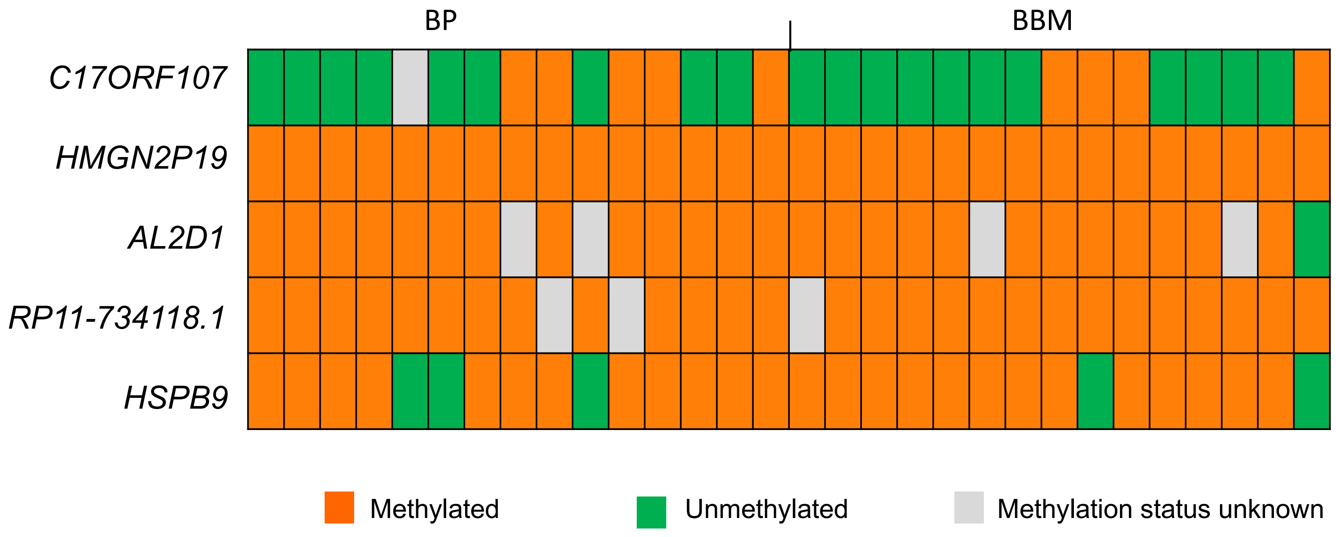

Supplementary Figure 6

Methylation status of genes validated by CoBRA.

Methylation status of genes that did not show differential methylation between BP and BBM therefore these genes were excluded for further analyses.

Supplementary Figure 7

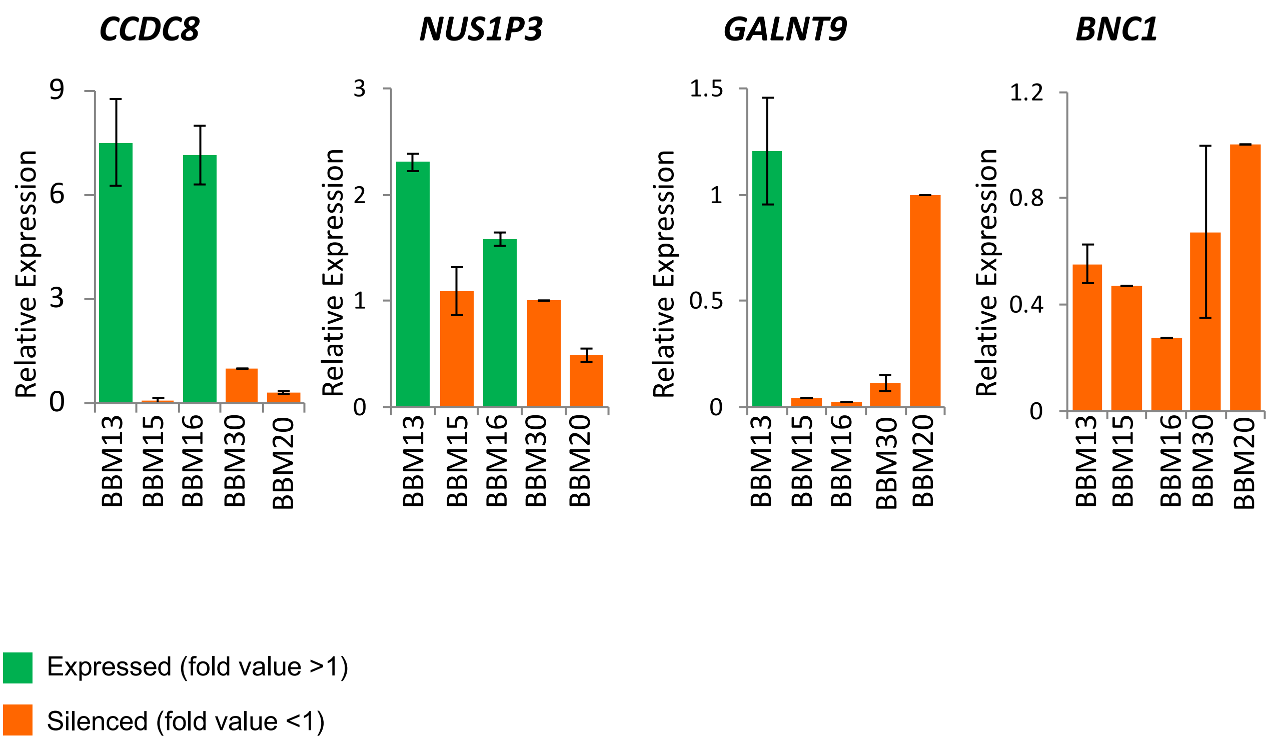

Supplementary Figure 7

Expression status of genes validated by CoBRA.

Expression status (QRT-PCR) of *CCDC8*, *NUS1P3*, *GALNT9* and *BNC1* in BBM tumours. Methylation is associated with reduced expression that correlates to their methylation status.

Supplementary Figure 8

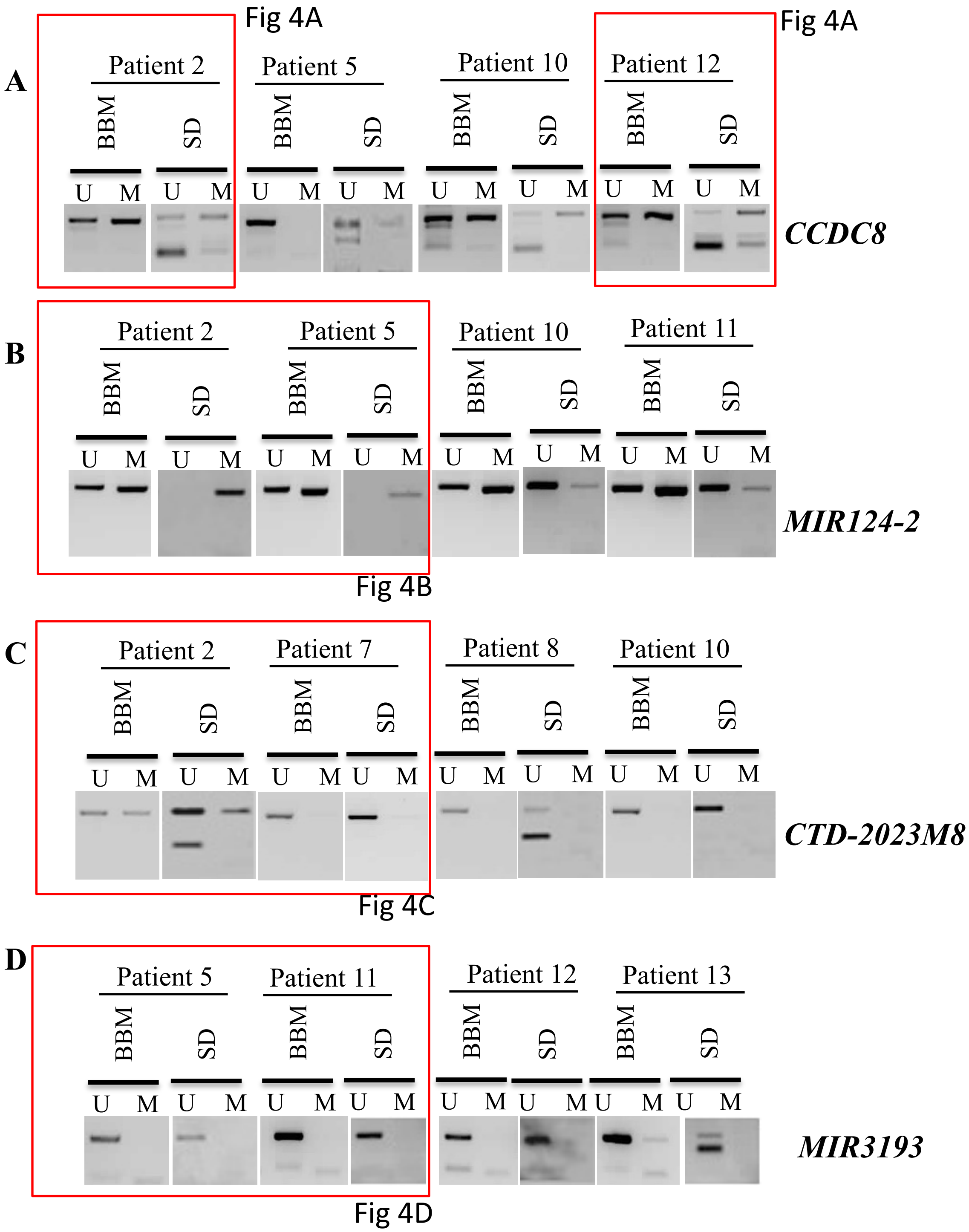

Supplementary Figure 8

Methylation status of candidate genes in BBM and tumour free circulating DNA (tfcDNA) in additional individual patients determined by Methylation Specific PCR (MSP).

(A, B) Methylation status of candidate genes denoting early events (*CCDC8* and *MIR124-2*) and (C, D) late events (*CTD-2023M8* and *MIR3193*) in BBM and their corresponding tumour free circulating DNA in individual patients. BBM: Brain Metastases, SD: Serum DNA, U: Unmethylation Specific PCR; USP, M: Methylation Specific PCR; MSP, *: Methylated samples.

Supplementary Figure 9

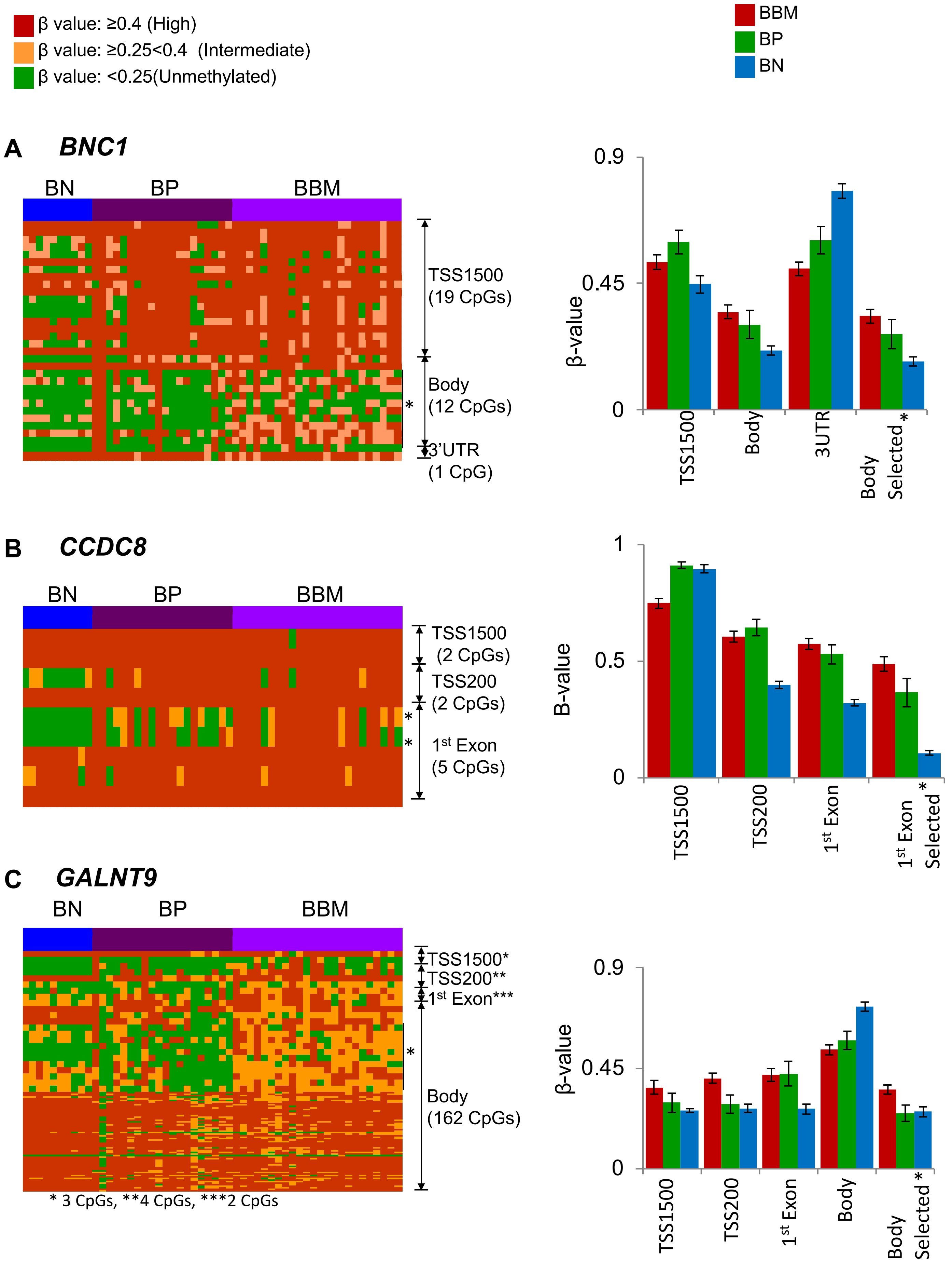

Supplementary Figure 9

Global methylation pattern of candidate genes

(A) *BNC1,* (B) *CCDC8 and* (C) *GLANT9,* showing the location of all CpG loci present on the 450K array relative to the transcription start of the gene and the differing methylation levels for each loci in BBM, BP and BN. * denotes an individual differentially methylated CpG that was resulted from initial screening of 450K-array data between BN, BP and BBM.

Supplementary Figure 10

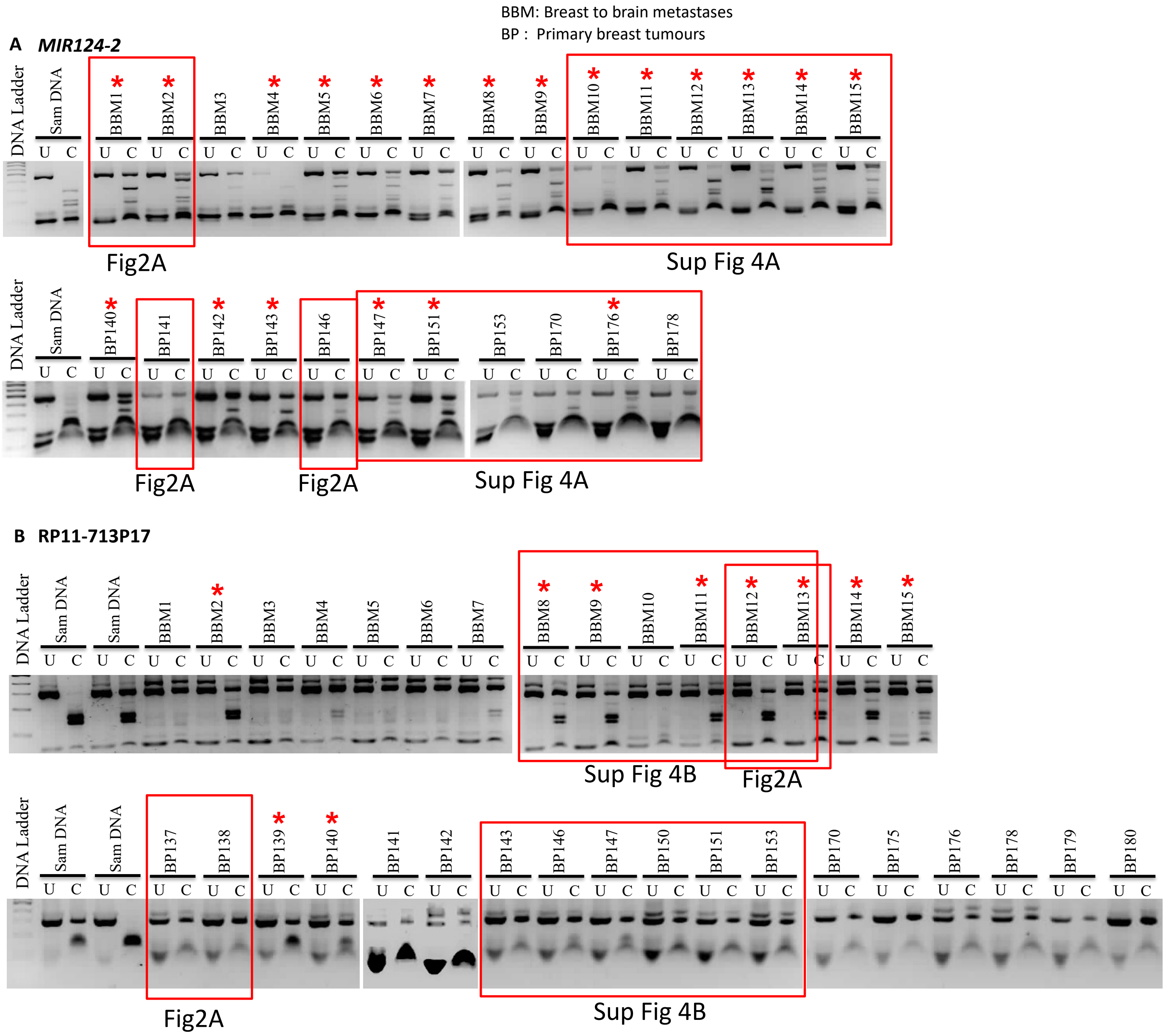

Supplementary Figure 10 continued………

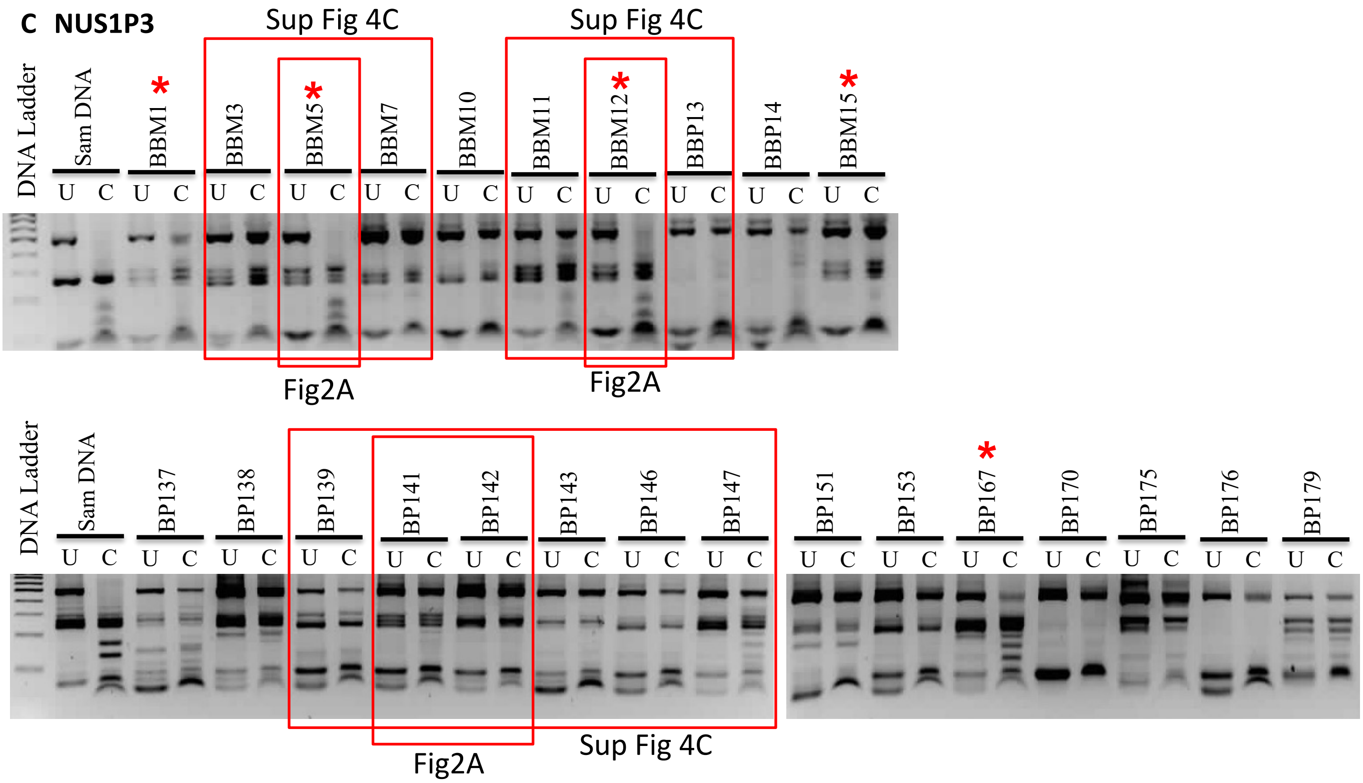

Supplementary Figure 10

Full (raw) CoBRA images for genes frequently methylated in BBM

Full CoBRA images for methylation analyses of additional samples in primary breast tumors (BP) and breast to brain metastases samples (BBM) on genes which are frequently methylated in BBM compared to BP. Red boxes indicate lanes used in the main manuscript figures.

Supplementary Figure 11

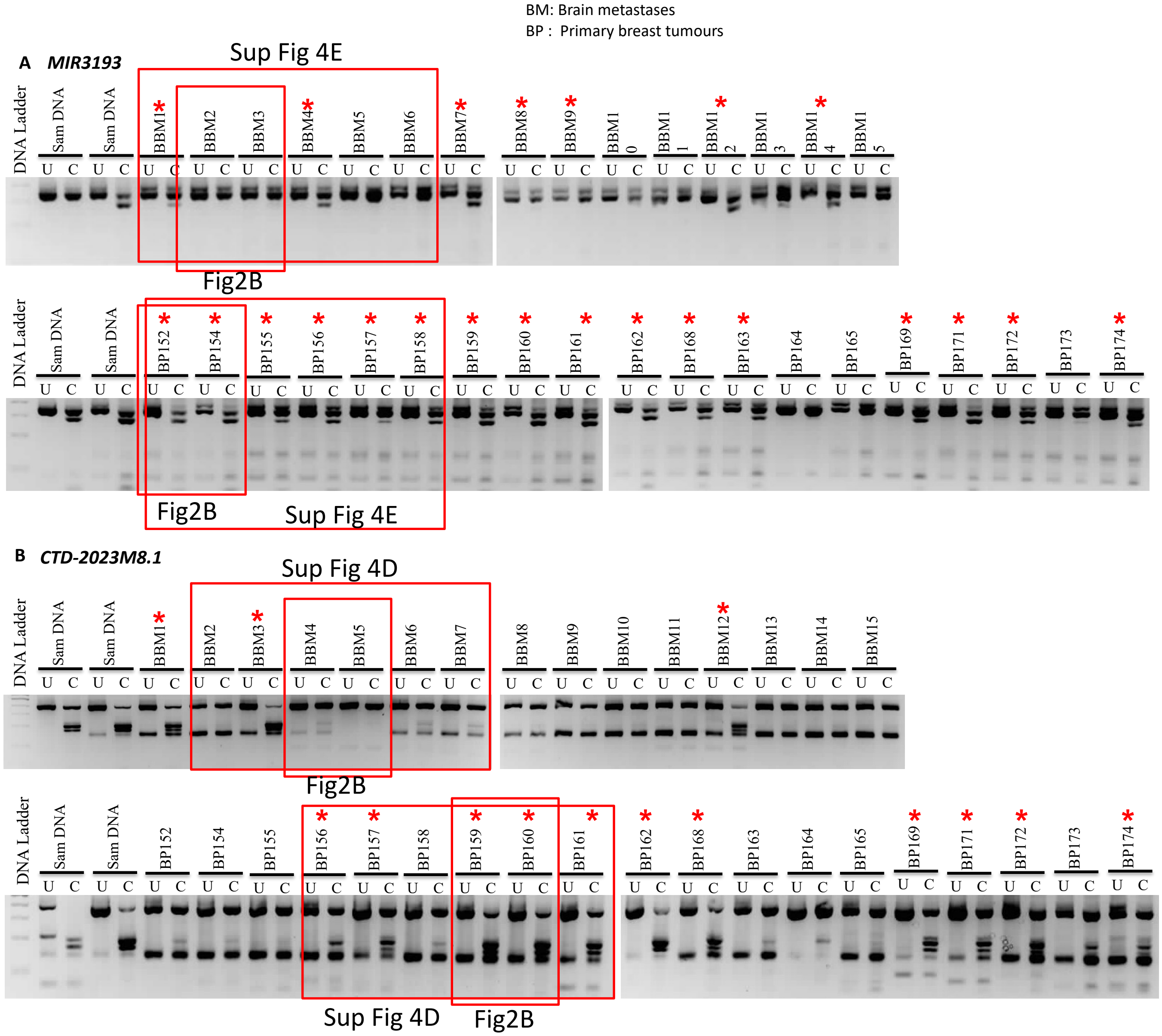

Supplementary Figure 11 continued………

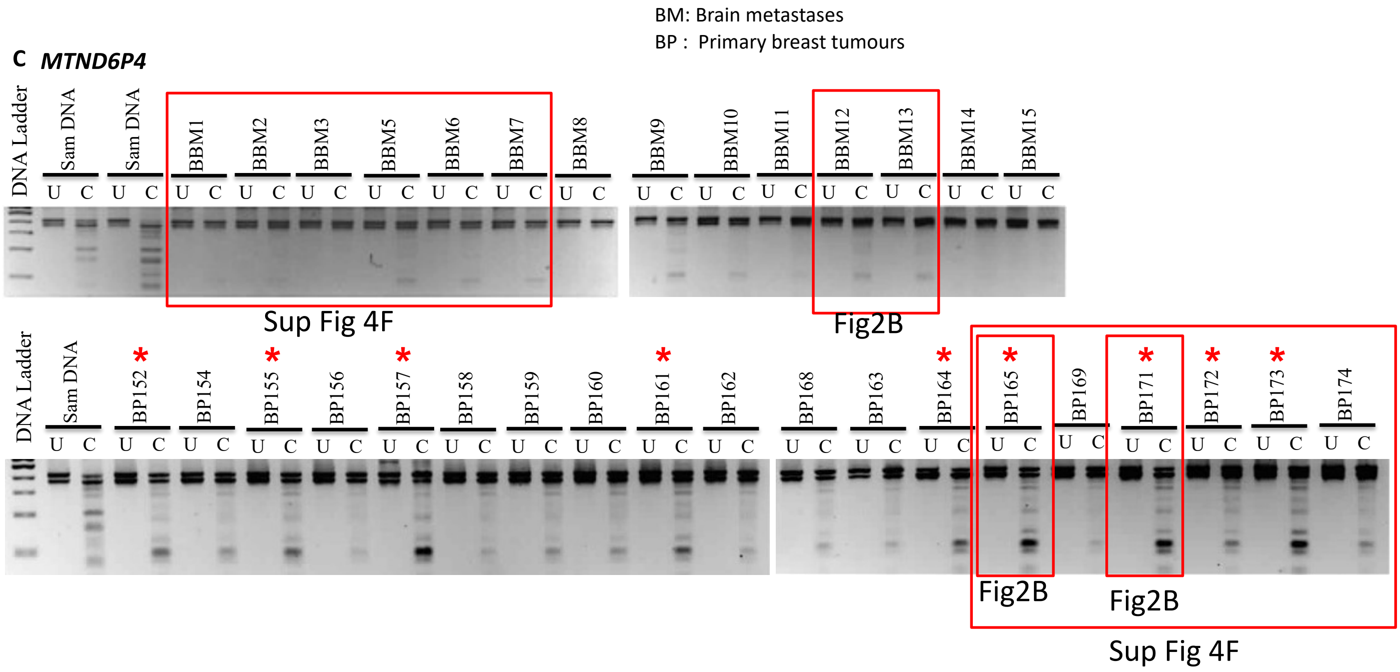

Supplementary Figure 11

Full (raw) CoBRA images for genes frequently methylated in primary breast tumors

Full CoBRA images for methylation analyses of additional samples in primary breast tumour samples (BP) and Breast to brain metastases (BBM) samples on genes which are frequently methylated in BP compared to BBM. Red boxes indicate lanes used in the main manuscript figures.

Supplementary Figure 12

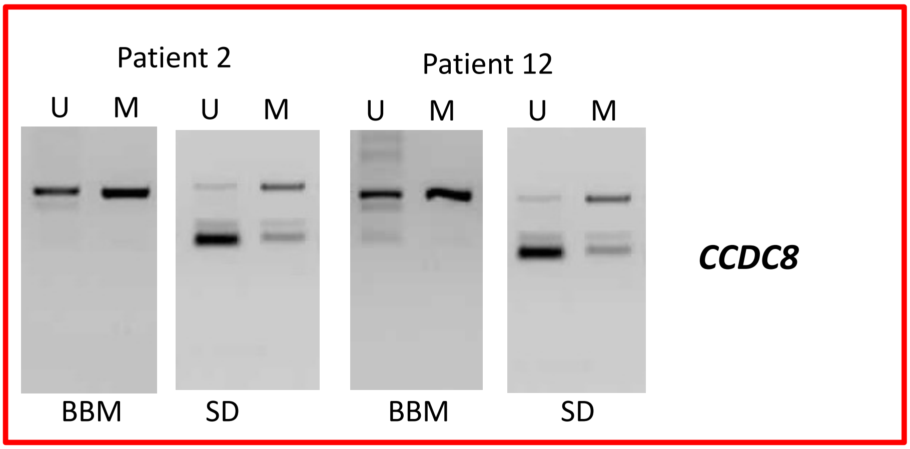

Figure 4A

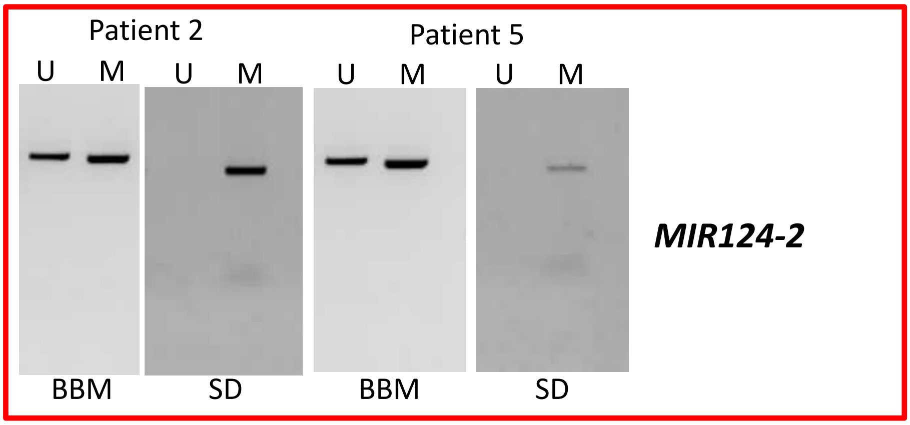

Figure 4B

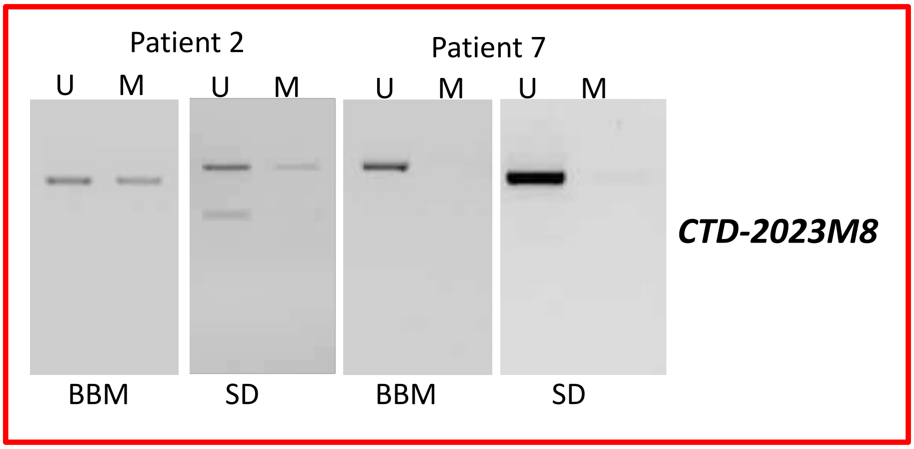

Figure 4C

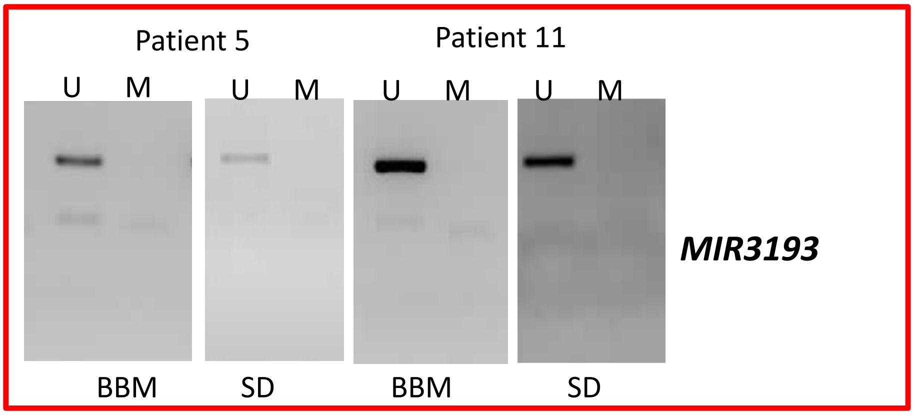

Figure 4D

Supplementary Figure 12

Methylation status of candidate genes in BBM and their corresponding serum DNA (raw gel images) related to main Figure 4.

Raw Methylation specific PCR (MSP images for (A) *CCDC8,* **(B)** MIR124-2, **(C)** *CTD-2023M8.1* and **(D)** *MIR3193*
